# Instrumental Cases

**Published:** 1883-10

**Authors:** 


### Instrument Cases.

In a long and interesting article by Dr. Landolt, in *Archives d' Ophthalmologie*, for July and August, in which he details carefully the instruments required in surgical operations on the eye, he makes reference to the box or case in which instruments are kept, which, in its language, is equally applicable to all surgical instrument cases. He says: "Formerly (and frequently to-day) instrument cases were generally of wood, covered with leather, and lined with velvet. We cannot deny that a box of that char, acter, handsomely finished, in Turkish morocco, ornamented with metallic corners and plates, lined with some rich material of bright color, with rows of glistening instruments arranged in close order, is a very pleasing sight (to the surgeon). But this bright picture has its shadows; the handsome velvet retains dirt too readily, and the luxurious interior of the box is absolutely opposed to a radical cleaning, and to an antisepsis in harmony with the doctrines of modern surgery. The simple box of wood has now come into vogue, and we have seen them of such handsome wood and superior workmanship as to rival their elders. They are made of wal. nut, mahogany, ebony, and rosewood. Walnut is the most solid, ebony the handsomest and dearest. Mahogany is very solid and gains in beauty with time. Foreign woods are sold by weight. Merchants frequently soak their woods in water to increase the weight and price to the detriment of their value. A wooden box should be sufficiently well made to be plunged with impunity into an antiseptic solution, and to sustain thorough cleanings with brush and sponge."

